# Detection and Prediction of Bioprosthetic Aortic Valve Degeneration

**DOI:** 10.1016/j.jacc.2018.12.056

**Published:** 2019-03-19

**Authors:** Timothy R.G. Cartlidge, Mhairi K. Doris, Stephanie L. Sellers, Tania A. Pawade, Audrey C. White, Renzo Pessotto, Jacek Kwiecinski, Alison Fletcher, Carlos Alcaide, Christophe Lucatelli, Cameron Densem, James H.F. Rudd, Edwin J.R. van Beek, Adriana Tavares, Renu Virmani, Daniel Berman, Jonathon A. Leipsic, David E. Newby, Marc R. Dweck

**Affiliations:** a British Heart Foundation Centre for Cardiovascular Science, University of Edinburgh, Edinburgh, United Kingdom; b Edinburgh Imaging Facility, Queen’s Medical Research Institute, University of Edinburgh, Edinburgh, United Kingdom; c Department of Radiology, St. Paul’s Hospital, University of British Columbia, Vancouver, British Columbia, Canada; d Department of Cardiology, Papworth Hospital NHS Foundation Trust, Cambridge, United Kingdom; e Division of Cardiovascular Medicine, University of Cambridge, Cambridge, United Kingdom; f CVPath Institute, Gaithersburg, Maryland; g Cedars-Sinai Heart Institute, Los Angeles, California

**Keywords:** aortic valve replacement, bioprosthetic valve degeneration, calcification, histology, positron emission tomography, CT, computed tomography, PET, positron emission tomography, TBR, target-to-background ratio

## Abstract

**Background:**

Bioprosthetic aortic valve degeneration is increasingly common, often unheralded, and can have catastrophic consequences.

**Objectives:**

The authors sought to assess whether ^18^F-fluoride positron emission tomography (PET)-computed tomography (CT) can detect bioprosthetic aortic valve degeneration and predict valve dysfunction.

**Methods:**

Explanted degenerate bioprosthetic valves were examined ex vivo. Patients with bioprosthetic aortic valves were recruited into 2 cohorts with and without prosthetic valve dysfunction and underwent in vivo contrast-enhanced CT angiography, ^18^F-fluoride PET, and serial echocardiography during 2 years of follow-up.

**Results:**

All ex vivo, degenerate bioprosthetic valves displayed ^18^F-fluoride PET uptake that colocalized with tissue degeneration on histology. In 71 patients without known bioprosthesis dysfunction, 14 had abnormal leaflet pathology on CT, and 24 demonstrated ^18^F-fluoride PET uptake (target-to-background ratio 1.55 [interquartile range (IQR): 1.44 to 1.88]). Patients with increased ^18^F-fluoride uptake exhibited more rapid deterioration in valve function compared with those without (annualized change in peak transvalvular velocity 0.30 [IQR: 0.13 to 0.61] vs. 0.01 [IQR: −0.05 to 0.16] ms^−1^/year; p < 0.001). Indeed ^18^F-fluoride uptake correlated with deterioration in all the conventional echocardiographic measures of valve function assessed (e.g., change in peak velocity, r = 0.72; p < 0.001). Each of the 10 patients who developed new overt bioprosthesis dysfunction during follow-up had evidence of ^18^F-fluoride uptake at baseline (target-to-background ratio 1.89 [IQR: 1.46 to 2.59]). On multivariable analysis, ^18^F-fluoride uptake was the only independent predictor of future bioprosthetic dysfunction.

**Conclusions:**

^18^F-fluoride PET-CT identifies subclinical bioprosthetic valve degeneration, providing powerful prediction of subsequent valvular dysfunction and highlighting patients at risk of valve failure. This technique holds major promise in the diagnosis of valvular degeneration and the surveillance of patients with bioprosthetic valves. (18F-Fluoride Assessment of Aortic Bioprosthesis Durability and Outcome [18F-FAABULOUS]; NCT02304276)

The implantation of bioprosthetic heart valves is increasing rapidly due to patient preference, the increasing prevalence of valve disease in an aging population, and the emergence of transcatheter aortic valve implantation 1, 2, 3, 4. In the United States, 90,000 surgical aortic valve replacements are performed annually, with over three-quarters incorporating bioprosthetic valves (3). In addition, >80,000 transcatheter aortic valve implantation procedures have been performed since U.S. Food and Drug Administration approval in 2011 (4). Given the finite lifespan of these valves and the rapid expansion of the population of patients requiring regular surveillance, bioprosthetic valve degeneration will become a major cause of cardiovascular morbidity and health care burden over the coming decades.

The pathophysiology of bioprosthetic valve degeneration is poorly understood. Although calcification appears to contribute to both progressive valve narrowing and leaflet tears 5, 6, 7, noninvasive methods for detecting this process have been lacking, and the triggers of valve degeneration and calcification are unknown. Current standard of care relies on serial echocardiography and clinical assessment aimed at detecting the valve dysfunction that occurs only toward the end stages of the degeneration process. Unfortunately, many patients present in extremis with unheralded valve failure due to rapid-onset valvular obstruction or regurgitation, with repeat operation a high-risk undertaking. Indeed, emergency repeat aortic valve replacement surgery is associated with a mortality of 22.6% compared with 1.4% for elective repeat surgery (8). Detection of bioprosthetic valve degeneration is therefore highly desirable, allowing at-risk patients to be identified early, offered close tailored monitoring, and optimized timing of repeat elective intervention, thereby avoiding potentially catastrophic valve failure.

^18^F-fluoride positron emission tomography (PET) has recently been used to image tissue calcification activity in a range of cardiovascular diseases 9, 10, 11, 12. ^18^F-fluoride preferentially binds to areas of developing microcalcification indicative of tissue degeneration (13) that precede the macrocalcification detectable by computed tomography (CT) 14, 15. Given that calcification is one of the key pathological processes underlying bioprosthetic valve degeneration, we hypothesized that increased ^18^F-fluoride uptake would identify prosthetic valve degeneration and predict subsequent deterioration in bioprosthetic valve function.

## Methods

### Ex vivo assessment of degenerated bioprosthetic aortic valves

Explanted degenerated aortic valve bioprostheses were obtained with written consent from patients undergoing repeat surgical aortic valve replacement for bioprosthetic valve failure. Valves were weighed, photographed, and their macroscopic features documented. Ex vivo micro–PET-CT was performed with a nano–PET-CT scanner (Mediso, Budapest, Hungary) and x-ray microtomograph (Bruker, Kontich, Belgium) before undergoing histological evaluation (Online Appendix).

### Clinical study design and population

Patients over 40 years of age who had undergone previous surgical aortic valve replacement using a bioprosthetic valve were prospectively recruited into a single-center cohort study according to defined inclusion and exclusion criteria (Online Figure 1). Participants were recruited if they were under routine clinical review and had: 1) known evidence of bioprosthetic valve failure on echocardiography and had been referred for repeat aortic valve intervention 16, 17; or 2) no known evidence of valve dysfunction or degeneration. Written informed consent was obtained from all participants. The study (NCT02304276) was conducted in accordance with the Declaration of Helsinki and was approved by an NHS Scotland Research Ethics Committee (14/SS/1049) and the Administration of Radioactive Substances Advisory Committee.

### Study assessments and data collection

All participants underwent baseline clinical assessment including Doppler and 2-dimensional echocardiography, noncontrast CT calcium scoring, contrast-enhanced CT angiography, and in vivo ^18^F-fluoride PET (Online Appendix, Online Table 1). Participants were invited to return annually for 2 years for repeat clinical assessments and echocardiography to assess changes in bioprosthesis performance. Definitions of bioprosthetic degeneration and dysfunction vary, but in this study, we used definitions from both contemporary international guideline criteria 16, 17 and a recent expert consensus statement (18) (Online Appendix). Bioprosthetic valve failure was defined as the development of severe hemodynamic valve dysfunction combined with patient symptoms, the need for redo valve intervention, or valve-related death (19). Patients were followed up for the development of valve failure beyond the 2-year timeframe using electronic patient record data.

### PET and CT image analysis

Fused PET and contrast-enhanced CT images were reconstructed in diastole, coregistered and reoriented to provide en face images of the bioprosthetic valve with corresponding long-axis views (Online Figure 2). Images were analyzed by 2 experienced PET-CT observers (T.R.G.C. and M.R.D.). Using pre-specified criteria, CT scans were adjudicated to be abnormal if there was pannus (circumferential low-attenuation [noncalcific] material with radial thickness ≥2 mm and encroachment on the valve cusps) 16, 20, noncalcific leaflet thickening (focal areas of low attenuation [30 to 200 Hounsfield Units (HU)] cusp thickening ≥2 mm visualized in at least 2 planes) 20, 21, or leaflet calcification (calcium >500 HU localized to valve cusp in ≤2 planes) (22). Leaflet calcification was further subdivided into spotty calcification if maximum diameter was <3 mm and large calcification if maximum diameter was ≥3 mm (23). ^18^F-fluoride uptake was quantified primarily using the most-diseased-segment mean target-to-background ratio (TBR) method in keeping with prior studies of the native valve, although other measures were also reported (24). PET scans were adjudicated to be abnormal if increased ^18^F-fluoride uptake (threshold TBR >1.3) (11) was observed originating from the valve cusps on both en face and long-axis views (Online Appendix).

### Statistics and data analysis

Baseline characteristics are reported as number (percentages) for categorical variables and mean ± SD or median (interquartile range) for continuous variables depending on whether variables were normally distributed. Categorical data were compared using chi-square or Fisher exact tests. Continuous variables were log-transformed [log_N_(x+1) for positive values and −log_N_(−x+1) for negative values] where not normally distributed. The point-biserial correlation coefficient was used to measure the strength and direction of the association between 1 dichotomous variable and 1 continuous variable. One-way analysis of variance was used to compare continuous data across multiple factors, with post hoc analysis using the Bonferroni test where appropriate. The Student’s *t*-test or Mann-Whitney *U* test were used to compare continuous outcomes between 2 independent groups depending on whether they were normally distributed. The Student’s *t*-test or Wilcoxon signed rank test were used to compare paired variables. Two-tailed Pearson’s correlation analysis was performed to investigate the relationship between ^18^F-fluoride uptake and echocardiographic measures of valve function. Multivariable analysis was performed to assess the predictors of deterioration in bioprosthetic valve dysfunction (annualized change in peak velocity after 2 years). Statistical analysis was undertaken using IBM SPSS Statistics version 23 software (IBM, Armonk, New York), and significance was taken at the 2-sided 5% level (p < 0.05).

## Results

### Explanted degenerate bioprosthetic aortic valves

**Figure 1:**
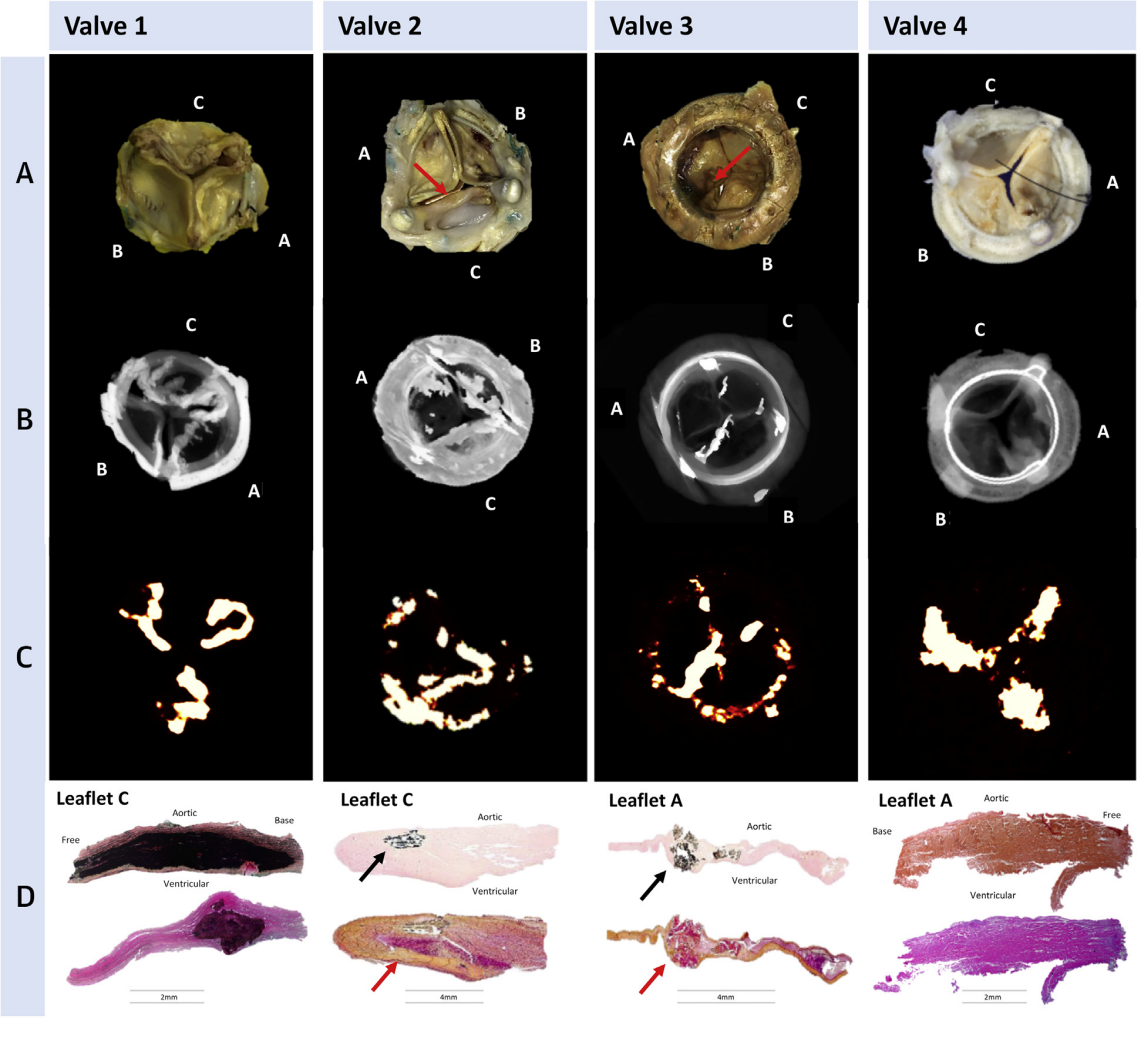
Ex Vivo Degenerated Bioprosthetic Aortic Valves: Macroscopic Appearances, Micro-CT, Micro-PET, and Histology **(Row A)** Macroscopic visual appearances of failed and explanted bioprosthetic valves. **(Row B)** CT en face images of the valves. **(Row C)** PET en face images demonstrating increased ^18^F-fluoride uptake in all valves. **(Row D)** Histology staining of sections taken from valve leaflet as indicated, with von Kossa (**top row**, calcium appears black), Movat Pentachrome (**bottom row**, valves 1 and 4), and hematoxylin and eosin (**bottom row**, valves 2 and 3) stains. All 4 degenerate bioprostheses demonstrate increased ^18^F-fluoride uptake in the valve leaflets. In valve 1, this uptake corresponds to gross leaflet calcification observed macroscopically and on CT images with confirmation on histology (extensive black staining). In valve 2, increased ^18^F-fluoride uptake is observed in association with fibrotic leaflet thickening and pannus **(red arrows)** with associated calcification **(black arrows)** observed macroscopically and on CT with confirmation on histology. In valve 3, increased ^18^F-fluoride uptake is observed at the site of valve leaflet thrombus **(red arrow)** observed macroscopically at the base of leaflet 1, with confirmation of thrombus **(red arrow)** and colocalized calcification **(black arrow)** on histology. In valve 4, extensive ^18^F-fluoride uptake is observed in the absence of calcification on CT and histology but instead in areas of leaflet thickening, marked fluid insudation, and disrupted collagen architecture. CT = computed tomography; PET = positron emission tomography.

Fifteen failed explanted bioprosthetic aortic valves were obtained (Online Appendix) for ex vivo investigation. Micro-CT detected leaflet calcification in 13 valves, which was confirmed on histology. By contrast, all 15 valves demonstrated ^18^F-fluoride leaflet uptake that correlated with a range of histological markers of bioprosthetic tissue degeneration. In particular, ^18^F-fluoride activity detected both micro- and macrocalcific deposits within the valve leaflets that colocalized predominantly with regions of pannus (fibrous thickening) and thrombus formation on histology. However, ^18^F-fluoride uptake was additionally observed in the absence of calcification on histology at sites of leaflet thickening, fluid insudation, and disrupted collagen architecture (Figure 1).

### Clinical cohort study population

Eighty participants were recruited to the clinical cohort study and underwent in vivo PET-CT imaging, although 2 patients were unable to complete the baseline scan (claustrophobia) and were excluded. The remaining 78 patients were 75 ± 7 years of age, with a range of bioprosthetic valve models (Online Tables 2 and 3), a high prevalence of coronary artery disease, and associated risk factors (Table 1).

**Table 1:** Baseline Characteristics of the Clinical Study Population Undergoing in Vivo PET and CT Imaging

	Patients With Valve Failure	Patients Without Known Valve Degeneration				
		All	1 Month	2 Year	5 Year	10 Year
Clinical						
Subjects	6	71	9	22	20	20
Female	2 (33.3)	38 (46.5)	5 (55.6)	8 (36.4)	10 (50.0)	10 (50.0)
Age, yrs	81.3 ± 3.2	73.9 ± 7.0	73.4 ± 9.0	73.0 ± 5.4	72.7 ± 5.1	76.4 ± 8.9
Body mass index, kg/m^2^	27.5 ± 4.5	26.9 ± 5.6	27.8 ± 4.7	27.7 ± 4.8	27.8 ± 5.1	24.8 ± 6.8
Systolic blood pressure, mm Hg	144.8 ± 18.0	153.8 ± 21.9	152.4 ± 21.2	149.8 ± 22.1	158.1 ± 15.2	154.5 ± 27.8
Diastolic blood pressure, mm Hg	58.0 ± 8.4	79.0 ± 11.0	82.0 ± 11.4	81.3 ± 12.0	78.6 ± 7.4	75.4 ± 12.6
Heart rate, beats/min	72.2 ± 17.0	71.2 ± 11.8	79.8 ± 10.0	69.7 ± 11.6	73.3 ± 13.4	66.6 ± 8.9
Medical history						
Hypertension	4 (66.7)	50 (70.4)	8 (88.9)	16 (72.7)	14 (70.0)	12 (60.0)
Coronary artery disease	4 (66.7)	29 (40.9)	5 (55.6)	7 (31.8)	6 (30.0)	11 (55.0)
Coronary bypass surgery	3 (50.0)	23 (32.4)	2 (22.2)	7 (31.8)	5 (25.0)	9 (45.0)
Diabetes	1 (16.7)	4 (5.6)	0 (0.0)	0 (0.0)	2 (10.0)	2 (10.0)
Hypercholesterolemia	5 (83.3)	55 (77.5)	8 (88.9)	18 (81.8)	13 (65.0)	16 (80.0)
Current smoker	0 (0.0)	8 (11.4)	0 (0.0)	1 (4.6)	3 (15.0)	4 (20.0)
Ex-smoker	3 (50.0)	29 (40.8)	4 (44.4)	10 (45.5)	7 (35.0)	8 (40.0)
Medication						
Aspirin	4 (66.7)	51 (71.8)	7 (77.8)	18 (81.8)	12 (60.0)	14 (70.0)
Clopidogrel	1 (16.7)	9 (12.7)	1 (11.1)	2 (9.1)	4 (20.0)	2 (10.0)
Warfarin	2 (33.3)	4 (5.6)	0 (0.0)	1 (4.5)	3 (15.0)	0 (0.0)
Other anticoagulant	0 (0.0)	2 (2.8)	0 (0.0)	0 (0.0)	1 (5.0)	1 (5.0)
ACEi or ARB	0 (0.0)	39 (54.9)	6 (66.7)	12 (54.5)	10 (50.0)	11 (55.0)
Beta-blocker	2 (33.3)	32 (45.1)	4 (44.4)	13 (59.1)	7 (35.0)	8 (40.0)
Statin	4 (66.7)	50 (70.4)	6 (66.7)	15 (68.2)	14 (70.0)	14 (70.0)
Biochemistry						
Creatinine, μmol/l	102.2 ± 37.2	84.5 ± 23.4	90.3 ± 36.4	80.9 ± 17.8	89.1 ± 19.8	81.3 ± 25.4
eGFR, ml/min/1.73 m^2^	48.5 ± 13.0	58 ± 7.5	53.4 ± 11.8	59.8 ± 3.2	57.1 ± 6.8	59.2 ± 8.5
Electrocardiogram						
Sinus rhythm	4 (66.7)	64 (91.4)	9 (100.0)	20 (95.2)	18 (90.0)	17 (85.0)
Paced rhythm	0 (0.0)	4 (5.7)	0 (0.0)	0 (0.0)	1 (5.0)	3 (15.0)
Atrial fibrillation	2 (33.3)	2 (2.9)	0 (0.0)	1 (4.8)	1 (5.0)	0 (0.0)
LV hypertrophy	3 (50.0)	22 (32.4)	4 (44.4)	7 (33.3)	9 (45.0)	2 (11.1)
Strain pattern	3 (50.0)	14 (20.6)	4 (44.4)	2 (9.5)	6 (30.0)	2 (11.1)
Baseline echocardiography						
LV systolic dysfunction	4 (66.7)	11 (15.5)	1 (11.1)	3 (13.6)	4 (20.0)	3 (15.0)
Peak valve velocity, m/s	3.19 (2.84–4.47)	2.73 (2.38–3.07)	2.44 (2.27–2.75)	2.68 (2.28–2.82)	2.93 (2.55–3.40)	2.85 (2.36–3.13)
Mean valve gradient, mm Hg	20.6 (14.0–39.5)	15.0 (11.3–19.3)	13.0 (10.0–15.0)	13.9 (10.2–17.8)	18.5 (13.1–23.8)	17.2 (11.1–20.8)
Effective orifice area, cm^2^	0.52 (0.52–0.52)	1.13 (0.94–1.46)	1.33 (0.95–1.60)	1.14 (0.95–1.46)	1.03 (0.92–1.31)	1.20 (0.86–1.49)
Dimensionless velocity index	0.29 (0.20–0.42)	0.39 (0.33–0.44)	0.47 (0.38–0.51)	0.40 (0.36–0.43)	0.35 (0.31–0.40)	0.38 (0.31–0.46)
Prosthetic regurgitation, ≥ moderate	5 (83.3)	2 (2.8)	0 (0.0)	0 (0.0)	0 (0.0)	2 (10.0)
Acceleration time, ms	*	80.3 (75.2–87.4)	87.0 (74.0–93.0)	80.0 (75.0–85.0)	79.0 (75.0–86.0)	85.0 (76.7–94.0)
Acceleration time/LV ejection time	*	0.25 (0.24–0.28)	0.29 (0.25–0.31)	0.25 (0.24–0.27)	0.25 (0.23–0.27)	0.25 (0.22–0.28)
Computed tomography						
Abnormal findings	6 (100.0)	14 (19.7)	0 (0.0)	3 (13.6)	4 (20.0)	7 (30.0)
Spotty calcification	3 (50.0)	5 (7.0)	0 (0.0)	1 (4.5)	1 (5.0)	3 (15.0)
Large calcification	3 (50.0)	0 (0.0)	0 (0.0)	0 (0.0)	0 (0.0)	0 (0.0)
Noncalcific leaflet thickening	2 (33.3)	5 (7.0)	0 (0.0)	1 (4.5)	3 (15.0)	1 (5.0)
Pannus	1 (16.7)	7 (9.9)	0 (0.0)	2 (9.1)	0 (0.0)	5 (25.0)
^18^F-fluoride positron emission tomography						
Leaflet uptake	6 (100.0)	24 (33.8)	1 (11.1)	6 (27.2)	8 (40.0)	9 (45.0)
SUV _MDS max_	4.66 (3.16–7.57)	1.73 (1.42–2.02)	1.89 (1.42–1.99)	1.50 (1.31–1.80)	1.94 (1.39–2.26)	1.73 (1.54–2.34)
SUV _MDS mean_	3.50 (2.64–5.90)	1.48 (1.27–1.84)	1.59 (1.29–1.82)	1.31 (1.14–1.57)	1.72 (1.21–2.05)	1.44 (1.32–2.04)
TBR _MDS max_	3.61 (2.37–5.82)	1.31 (1.18–1.70)	1.26 (1.17–1.35)	1.26 (1.12–1.47)	1.52 (1.17–1.82)	1.47 (1.24–2.12)
TBR _MDS mean_	2.91 (2.10–4.09)	1.12 (1.04–1.51)	1.07 (1.06–1.18)	1.11 (1.00–1.31)	1.38 (1.01–1.62)	1.15 (1.06–1.87)

Values are n, n (%), mean ± SD, or median (interquartile range). *Incomplete data.

ACEi = angiotensin-converting enzyme inhibitor; ARB = angiotensin receptor blockade; CT = computed tomography imaging; eGFR = estimated glomerular filtration rate; LV = left ventricle; MDS = most diseased segment; PET = positron emission tomography; SUV = standardized uptake value; TBR = target-to-background ratio.

### Patients with suspected bioprosthetic valve failure

**Figure 2:**
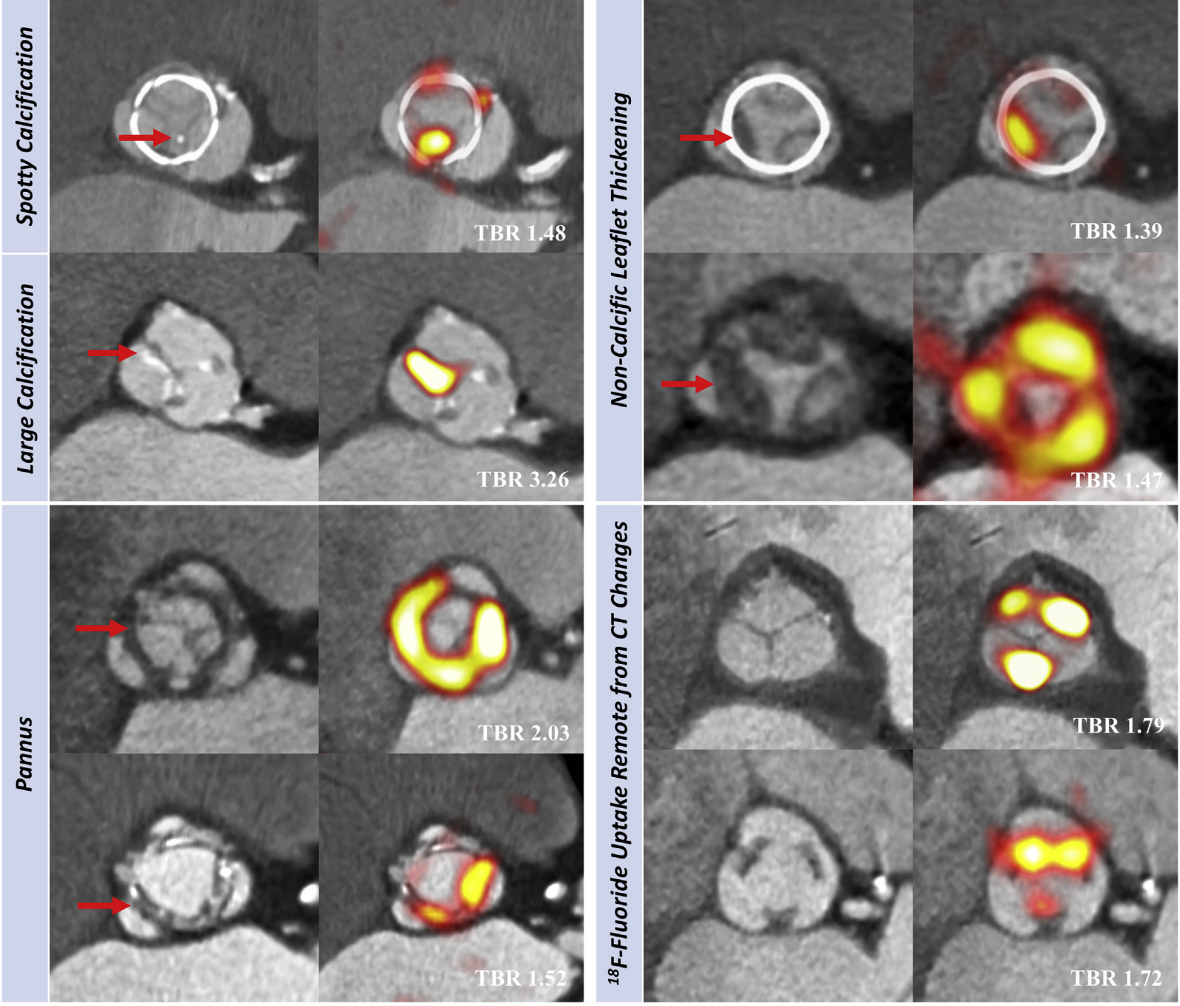
In Vivo ^18^F-fluoride PET and CT Imaging of Patients With Bioprosthetic Aortic Valves Baseline CT **(left)** and ^18^F-fluoride PET **(right)** images from patients with bioprosthetic aortic valves. En face CT images of aortic bioprosthetic valves showing spotty calcification and large calcification **(top left)**, circumferential pannus **(bottom left)**, and noncalcific leaflet thickening suggestive of thrombus **(top right)** (all abnormalities identified by **red arrows**). Hybrid en face PET-CT images in the same patients: increased bioprosthetic ^18^F-fluoride activity **(red/yellow areas)** is observed in each patient colocalizing with the CT abnormalities. ^18^F-fluoride activity was also commonly observed remote from leaflet changes on CT **(bottom right)**. Target-to-background (TBR) values are annotated on the hybrid PET-CT images in **white text**. Abbreviations as in Figure 1.

Seven participants were recruited to the cohort with suspected bioprosthetic valve failure. One subject proceeded to repeat surgical aortic valve replacement, 5 underwent valve-in-valve transcatheter valve implantation, and 1 subject was deemed to have severe patient–prosthesis mismatch according to guideline criteria and therefore not considered to have valve failure 16, 17. All 6 subjects with confirmed bioprosthetic valve failure demonstrated abnormalities on both CT and ^18^F-fluoride PET studies (Figure 2). CT revealed evidence of leaflet calcification in all patients (spotty calcification n = 3, large calcification n = 3) and less commonly noncalcific leaflet thickening (n = 2), circumferential pannus (n = 1), and distorted leaflet morphology (n = 1). Increased ^18^F-fluoride PET leaflet uptake was observed in the bioprosthetic valves of all these patients, and TBR values were nearly 3 times higher than in patients without known valvular dysfunction (TBR 2.91 [interquartile range (IQR): 1.75 to 4.09] vs. 1.12 [IQR: 1.04 to 1.51]; p < 0.001). In the 1 patient with severe patient–prosthesis mismatch, there was no CT abnormality or ^18^F-fluoride uptake.

### Patients without known bioprosthetic valve dysfunction

In the 71 patients without known bioprosthetic valve dysfunction, surgical aortic valve replacement had been conducted 1 month (n = 9), 2 years (n = 22), 5 years (n = 20), and >10 years (n = 20) previously. The function of these different-aged valves was generally within normal limits at baseline (peak velocity 2.76 ± 0.52 m/s, mean gradient 16.4 ± 7.0 mm Hg) (Table 1).

Contrast-enhanced CT images were not interpretable in 8 patients (11%) due to motion degradation and metallic blooming artefact from the valve struts. Fourteen subjects (19%) had 1 or more leaflet abnormalities on CT: spotty calcification (n = 5), noncalcific leaflet thickening (n = 5), and pannus (n = 7) (Figure 2).

PET scans were interpretable in all patients. Increased ^18^F-fluoride uptake was more common than abnormalities on CT and was seen in 24 patients (34%) (TBR 1.55 [IQR: 1.44 to 1.88]) (Figure 2, Online Figure 3). Similar to the ex vivo findings, increased ^18^F-fluoride uptake colocalized with areas of spotty calcification, noncalcific leaflet thickening (suggestive of thrombus), and pannus observed on the CT, but was also observed remote from CT abnormalities (Figure 2).

### Prediction of bioprosthetic valve dysfunction

Sixty-seven of the patients without established valve degeneration at baseline underwent repeat echocardiography at 2 years and demonstrated increased peak velocities compared with baseline (2.87 [IQR: 2.52 to 3.13] m/s vs. 2.73 [IQR: 2.38 to 3.07] m/s; p = 0.002). There was no association between hemodynamic progression and either the type or the age of the bioprosthesis (Online Table 4).

The 14 patients with abnormal CT findings appeared to demonstrate deterioration in bioprosthetic valve function after 2 years, but there was no statistical difference in disease progression compared with patients with a normal CT (change in peak velocity: 0.25 [IQR: −0.06 to 0.57] m/s vs. 0.10 [IQR: −0.04 to 0.31] m/s; p = 0.232).

By contrast, the 24 patients with increased ^18^F-fluoride uptake at baseline demonstrated clear evidence of deteriorating bioprosthesis function after 2 years, whereas patients without uptake displayed no change in valve function (change in peak velocity: 0.30 [IQR: 0.13 to 0.61] vs. 0.01 [IQR: −0.05 to 0.16] m/s/year; p < 0.001). Similarly, valves demonstrated progressive hemodynamic deterioration during follow-up on moving across tertiles of ^18^F-fluoride uptake (Online Figure 3). Moreover, baseline ^18^F-fluoride uptake correlated strongly with all echocardiographic measures of hemodynamic progression regardless of the method used to quantify ^18^F-fluoride uptake (Table 2).

**Table 2:** Correlation Between Bioprosthetic ^18^F-fluoride Uptake and Subsequent Deterioration in Valve Function by Echocardiography

Annualized Change	SUV Mean (Log_N_)	SUV Max (Log_N_)	TBR Mean (Log_N_)	TBR Max (Log_N_)
Peak valve velocity, Log_N_	r = 0.58 p < 0.001*	r = 0.62 p < 0.001*	r = 0.72 p < 0.001*	r = 0.72 p < 0.001*
Mean valve gradient, Log_N_	r = 0.42 p < 0.001*	r = 0.45 p = 0.001*	r = 0.52 p < 0.001*	r = 0.52 p < 0.001*
Effective orifice area, cm^2^/yr	r = −0.45 p < 0.001*	r = −0.52 p < 0.001*	r = −0.57 p < 0.001*	r = −0.60 p < 0.001*
Doppler velocity index	r = −0.46 p < 0.001*	r = −0.51 p < 0.001*	r = −0.59 p < 0.001*	r = −0.60 p < 0.001*

In 71 volunteers with bioprosthetic aortic valves without known valve dysfunction, baseline ^18^F-fluoride uptake measured by positron emission tomography using any conventional method of quantification (SUV or TBR) was associated with subsequent deterioration in each echocardiographic measure of valve function. Note all values are based upon the most-diseased-segment approach. The TBR mean values were used as the primary comparison and are referred to elsewhere in the paper simply as the TBR abbreviation. *Statistically significant.

Abbreviations as in Table 1.

**Figure 3:**
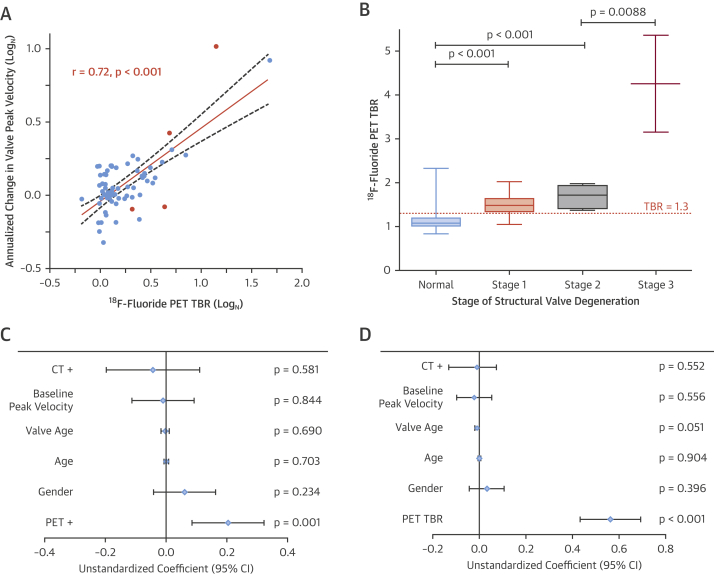
Baseline ^18^F-fluoride PET Uptake Predicts Subsequent Deterioration in Bioprosthetic Valve Function After 2 Years **(A)** A strong correlation was observed between baseline ^18^F-fluoride uptake in the bioprosthetic valves (TBR) and subsequent progression in bioprosthetic valve peak velocity (log transformation applied; r = 0.72; p < 0.001). **Orange dots** signify patients who developed new bioprosthetic valve regurgitation during follow-up. **(B)** ^18^F-fluoride uptake (**dashed orange line** represents threshold for increased ^18^F-fluoride uptake; TBR 1.3) in patients with different stages of structural valve degeneration after 2-year follow-up (stage 0: no significant change from post-implantation [n = 54]; stage 1: morphological abnormalities without significant hemodynamic changes [n = 9]; stage 2: new moderate stenosis and/or regurgitation [n = 5]; stage 3: new severe stenosis and/or severe regurgitation [n = 2]) (18) demonstrating incrementally higher uptake values with increasing severity of structural valve degeneration. **(C and D)** Forest plots of unstandardized coefficients (95% confidence intervals) from a multivariable linear regression analysis predicting change in bioprosthetic valve function (annualized change in peak velocity) during follow-up. When examining all relevant baseline characteristics, ^18^F-fluoride uptake was the only independent predictor of hemodynamic deterioration in valve function when used both as a dichotomous variable (PET+, TBR >1.3) **(C)** and as a continuous variable (TBR) **(D)**. CI = confidence interval; other abbreviations as in Figures 1 and 2.

**Figure 4:**
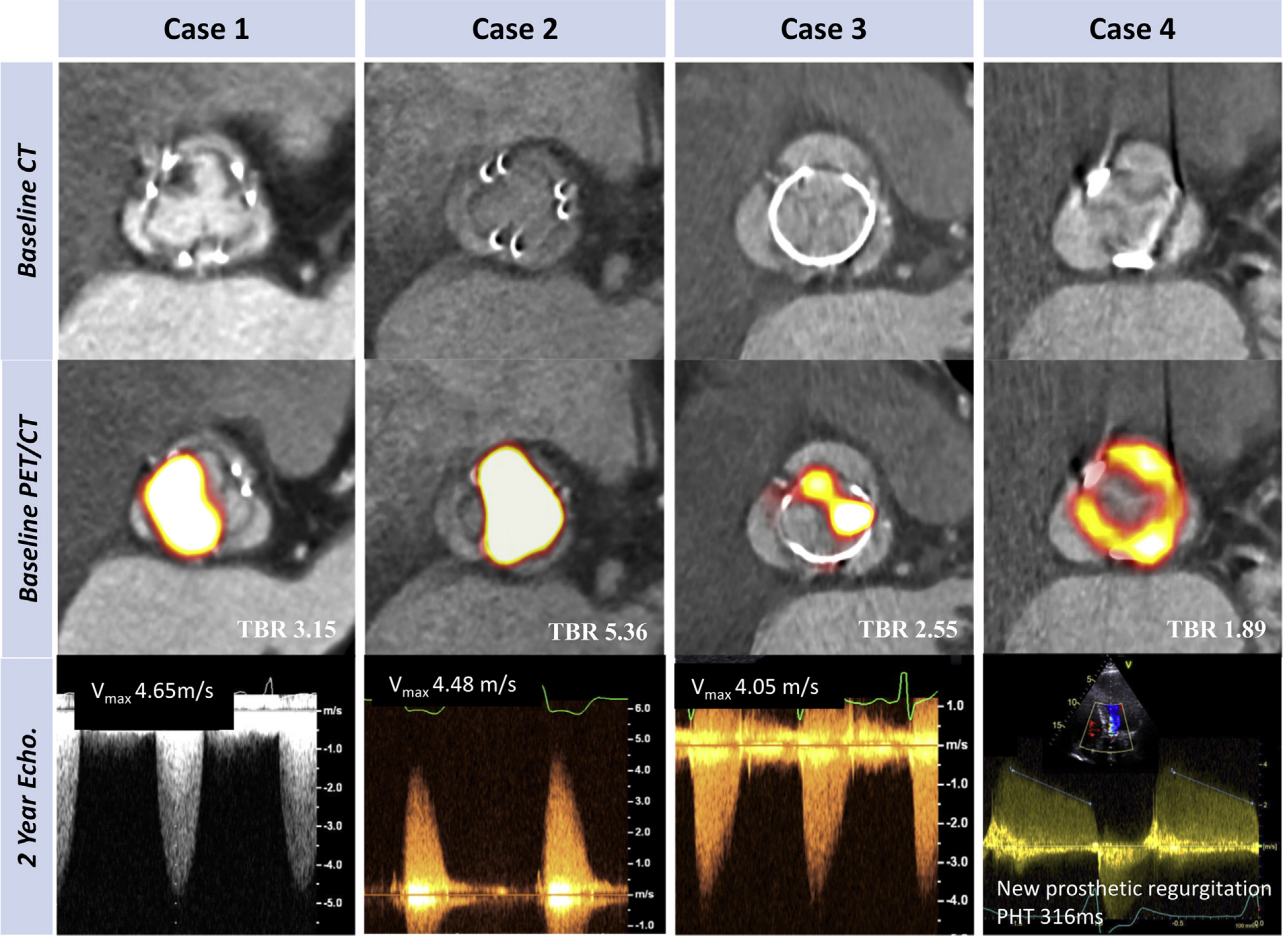
Case Illustrations: Baseline ^18^F-fluoride PET and CT Predict Imminent Failure of Bioprosthetic Function Cases 1 to 4 illustrate the utility of ^18^F-fluoride PET and CT in the prediction of deteriorating valve performance. None of the patients had known bioprosthetic degeneration at baseline. En face contrast-enhanced CT images **(top row)** demonstrate noncalcific leaflet thickening in case 1, but no clear structural CT changes in the remaining cases. Hybrid ^18^F-fluoride PET-CT images **(middle row)** demonstrate high-intensity ^18^F-fluoride activity in all the valves (TBR values in **white**). Doppler echocardiographic assessments of bioprosthetic valve function after follow-up **(bottom row)**. In each case, new valve dysfunction has developed with progression to severe obstruction in cases 1, 2, and 3 and new moderate/severe eccentric regurgitation in case 4 (pressure half time 316 ms with holodiastolic flow reversal in aorta). Patient #1 died from valve-related heart failure, Patient #2 required redo surgical valve replacement, Patient #3 remains under close surveillance, and Patient #4 is undergoing work-up for redo surgery.

On the basis of international guideline criteria 16, 17, 10 patients developed new bioprosthetic valve dysfunction during follow-up: 2 with valve regurgitation, 6 with valve stenosis, and 2 with mixed dysfunction. Median time from valve implantation to their assessment in the study was 7.5 (5 to 10) years. Of these patients, 5 had an abnormal baseline CT, whereas all 10 patients had increased ^18^F-fluoride uptake (TBR 1.89 [IQR: 1.46 to 2.59]), and this included the 7 patients with the highest TBR values in the cohort. Two proceeded to urgent valve reintervention, and another died of valve failure. Using the alternative criteria suggested by the recent international consensus statement (18), 16 patients met the definition of structural valve degeneration, 15 of whom demonstrated increased ^18^F-fluoride uptake (TBR 1.54 [IQR: 1.38 to 1.96] vs. 1.08 [IQR: 1.02 to 1.19]; p < 0.001). Seven patients fulfilled criteria for stage 2 or 3 structural valve degeneration, signifying the development of valve dysfunction, and they all exhibited increased baseline ^18^F-fluoride uptake (TBR 1.89 [IQR: 1.47 to 3.15]) (Figure 3). Moreover, ^18^F-fluoride activity increased in a stepwise fashion across these progressive stages of structural degeneration (TBR no degeneration: 1.08 [IQR: 1.02 to 1.19], stage 1: 1.48 [IQR: 1.34 to 1.64], stage 2: 1.72 [IQR: 1.42 to 1.94], stage 3: 4.23 [IQR: 3.15 to 5.36]; post hoc linear trend p < 0.001) (Figure 3). Two patients developed overt bioprosthetic valve failure during 2-year follow-up, and a further 2 patients developed valve failure on subsequent follow-up, all demonstrating high-intensity ^18^F-fluoride uptake (TBR 2.57 [IQR: 1.96 to 3.70]) (19) (Figure 4).

On univariable analysis, the only predictors of deterioration in bioprosthetic valve function (annualized change in bioprosthetic valve peak velocity) were current smoking habit (p = 0.047) and ^18^F-fluoride PET uptake, irrespective of whether the latter was considered as a categorical or continuous variable (both p < 0.001) (Online Table 4). On multivariable analysis incorporating age, sex, duration of valve implantation, baseline peak prosthetic valve velocity, and CT findings, ^18^F-fluoride uptake emerged as the only predictor of deterioration in bioprosthetic valve function (unstandardized coefficient 0.79 [95% confidence interval: 0.62 to 0.96]; p < 0.001) (Figure 3, Online Table 5).

Irrespective of the definition used 16, 17, 18, similar results were observed when the development of bioprosthetic valve dysfunction was considered as a categorical variable, with baseline ^18^F-fluoride uptake emerging each time as an independent predictor on multivariable analyses (international guideline criteria 16, 17: unstandardized coefficient 7.57 [standard error 2.66]; p = 0.004); expert consensus statement [18]: unstandardized coefficient 6.81 [standard error 2.92]; p = 0.02) (Online Table 6).

## Discussion

In this multimodality prospective imaging study, we have identified ^18^F-fluoride PET-CT as the first noninvasive technique capable of detecting early bioprosthetic valve degeneration and of predicting future valve dysfunction (Central Illustration). We provide extensive validation of the technique against state-of-the-art ex vivo imaging and histology. This consistently demonstrated increased ^18^F-fluoride uptake in each of the failed bioprosthetic aortic valves examined, with PET activity colocalizing to areas of calcification, pannus, thrombus, and disrupted tissue architecture on histology. When applied to patients in the clinical setting, ^18^F-fluoride PET identified early valve degeneration beyond the resolution of conventional assessments and predicted the development of new valvular dysfunction and overt valve failure within the 2-year follow-up period. Indeed, ^18^F-fluoride uptake was an independent predictor of deteriorating bioprosthetic valve performance, outperforming all other variables, including valve type and age, and echocardiographic and CT findings. ^18^F-fluoride PET-CT, therefore, provides a readily applicable measure of valve degeneration with the potential to transform how we monitor and treat the expanding population of patients living with bioprosthetic valves.

**Central Illustration:**
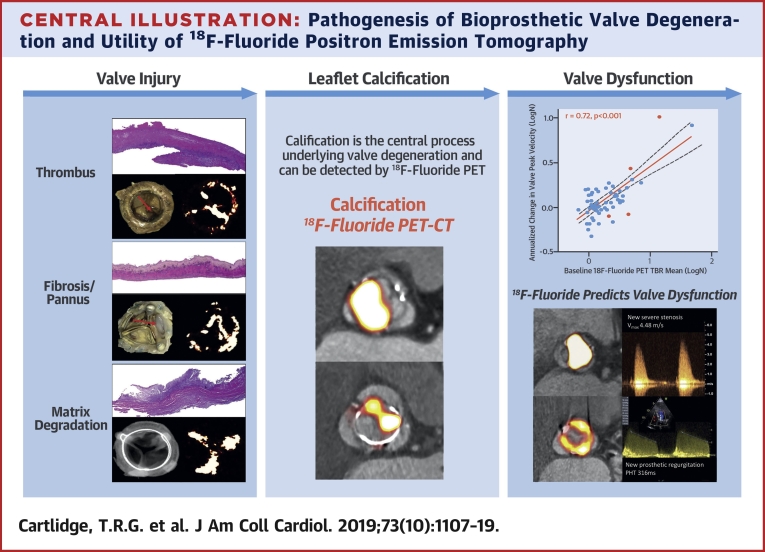
Pathogenesis of Bioprosthetic Valve Degeneration and Utility of ^18^F-Fluoride Positron Emission Tomography **(Left column)** Potential mechanisms of initial valve injury include leaflet thrombosis, fibrosis, and matrix degradation with expansion of the central proteoglycan layer, fluid insudation, and disruption of normal collagen architecture. **(Middle column)** Each appears to lead to bioprosthetic valve calcification as the final common pathway for valve degeneration that can be detected with ^18^F-fluoride PET imaging. **(Right column)** Baseline ^18^F-fluoride uptake correlates strongly with subsequent deterioration in valve function during follow-up (annualized change in peak transvalvular velocity: r = 0.72; p < 0.001) **(top)**. Two examples demonstrate high-intensity ^18^F-fluoride leaflet uptake at baseline in valves without previous evidence of degeneration. After follow-up, both bioprosthetic valves developed clear evidence of dysfunction: one with severe stenosis, and the other, regurgitation. PET = positron emission tomography.

Our study has several major strengths. This is a comprehensive multimodal imaging study using ultrasound, CT, and PET in patients with bioprosthetic aortic valves of varying implant ages. Uniquely, we have been able to correlate imaging findings with state-of-the-art ex vivo imaging and histological characterization of explanted valves, enabling us to validate our imaging technique and provide novel insights into the pathogenesis of bioprosthetic valve degeneration. Moreover, our systematic prospective study design has allowed us to confirm the utility of baseline ^18^F-fluoride PET in identifying patients at risk of developing future bioprosthetic dysfunction and overt valve failure.

### A clinical challenge

Although bioprosthetic valves offer important advantages over mechanical valves, they have only limited durability 25, 26. Indeed, bioprosthetic valve degeneration has become a major clinical issue with the growing rate of bioprosthetic valve implantation and improved long-term patient survival. Although international guidelines recommend serial echocardiography for the detection of bioprosthetic degeneration 16, 17, visualization of the valve is often poor due to acoustic artefacts from the prosthesis, limiting its sensitivity. This means that valve degeneration is frequently well advanced before clinically overt valve dysfunction is apparent. Moreover, valve obstruction often evolves rapidly, whereas the catastrophic development of valvular regurgitation due to leaflet tears is frequently unheralded and unpredictable. Novel techniques capable of detecting the earlier stages of valve degeneration and allowing personalized and better planned management strategies for patients with bioprosthetic valves are therefore highly desirable.

### Insights into the mechanism of bioprosthetic valve degeneration

We and others have used ^18^F-fluoride PET to assess cardiovascular calcification activity and tissue degeneration in a range of conditions including coronary heart disease, stroke, abdominal aortic aneurysms, and aortic stenosis 9, 10, 11, 12. We now extend these observations to bioprosthetic valves and confirm that ^18^F-fluoride localizes to regions of developing leaflet calcification on histology and high-resolution micro–PET-CT imaging. Calcification is believed to be the final common response to bioprosthetic valve injury and a major driver toward valve dysfunction, causing both progressive cusp stiffness and obstruction as well as leaflet fragility and tears. In this study, all bioprosthetic valves with established degeneration and valve failure demonstrated increased ^18^F-fluoride uptake. Moreover, in both our ex vivo and in vivo studies, we observed a close spatial interaction of calcification with both leaflet thrombosis and pannus, suggesting these may be potential upstream triggers. By contrast, a minority of bioprosthetic valves appear to degenerate without any histological evidence of calcification. In these cases, the remarkable histological features were gross leaflet thickening, fluid insudation, and disruption of normal collagen architecture. Interestingly, these valves also exhibited high levels of ^18^F-fluoride uptake. We hypothesize that up-regulation of matrix metalloproteinases may be implicated in the degradation of bioprosthetic leaflet tissue and that these proteins have the potential to bind ^18^F-fluoride 27, 28. Further investigation of the mechanism of uptake is required.

Recent reports have described focal noncalcific leaflet thickening as a marker of thrombus formation 20, 29. Although these areas can cause acute valve obstruction, more commonly, they do not result in any immediate hemodynamic disturbance, leading some to question their clinical relevance. In our study, we also observed low-attenuation noncalcific leaflet thickening on CT, suggestive of nonobstructive thrombus, that was associated with both increased ^18^F-fluoride activity and delayed deterioration in valve performance over the following 2 years. Similar findings were observed in explanted valves, where areas of thrombus colocalized with calcium on Von Kossa staining and exhibited increased ^18^F-fluoride PET activity. This raises the hypothesis that bioprosthetic valve thrombosis may act as one potential trigger to valve degeneration that might be prevented with prompt detection and anticoagulation. Interestingly, recent observational data have suggested that bioprosthetic durability may be enhanced by concomitant anticoagulation therapy (30).

### Clinical implications

Our findings have several major implications for clinical practice. First, in addition to micro- and macrocalcification, ^18^F-fluoride PET-CT can identify a range of degenerative processes including fibrosis, thrombosis, and leaflet degradation with disrupted collagen architecture and fluid insudation. This has provided important insights into the pathogenesis of bioprosthetic valve failure and highlights potential targets for novel therapies aimed at improving valve durability. Second, ^18^F-fluoride PET can detect and quantify the early stages of valvular degeneration and identify patients otherwise thought to have normal valves but who are in fact at high risk of subsequent valve failure. Given the high mortality associated with emergency repeat valve replacement (8), these patients are likely to benefit from both close tailored surveillance and early elective intervention before the onset of abrupt valve failure. Conversely, patients without PET uptake, who here demonstrated very stable valve function during follow-up, could undergo less intensive surveillance. Our data suggest that 5 years post-implantation may be an appropriate stage at which to offer a PET-CT scan and guide follow-up, with further studies needed. Third, ^18^F-fluoride PET-CT appears of value in determining the presence of valvular degeneration in cases where the diagnosis and clinical management is uncertain, for example in patients with suspected patient–prosthesis mismatch. Finally, ^18^F-fluoride PET-CT provides a readily applicable measure of valve durability that may prove useful in assessing novel prosthetic designs such as transcatheter valves before these are extended into younger and healthier patient populations.

### Study limitations

It was initially envisaged that serial CT calcium scoring of bioprosthetic aortic valves would also provide a marker of valve degeneration. However, in our hands, we observed major artefact related to motion and the valve frame that precluded accurate analysis of CT calcium scores. Leaflet pathology was instead more successfully identified by contrast-enhanced CT, where superior anatomic detail allowed differentiation between pannus ingrowth, thrombosis, or calcification, although a minority of scans still could not be accurately adjudicated. Alongside the robust information provided by ^18^F-fluoride PET this is an important advantage of the hybrid PET/CT technique.

Although our study population is relatively large compared with other cardiovascular PET studies, external validation in a larger study population with longer follow-up would be welcome. Future studies may wish to investigate ^18^F-fluoride activity in novel bioprostheses, in particular aortic and mitral transcatheter valves, as well as assessing whether the bioprosthetic ^18^F-fluoride signal is modifiable with adjuvant therapies, such as anticoagulation in patients with associated thrombus or drugs that modify calcium metabolism (e.g., bisphosphonates, denosumab).

## Conclusions

^18^F-fluoride PET-CT detects early bioprosthetic valve degeneration, providing powerful prediction of subsequent deterioration in valve performance and highlighting patients at imminent risk of valve failure. This novel imaging approach has the potential to transform our understanding of bioprosthetic valve failure and the way in which we monitor and treat patients with bioprosthetic valves.

Perspectives **COMPETENCY IN PATIENT CARE AND PROCEDURAL SKILLS:** ^18^F-fluoride PET-CT imaging can detect early bioprosthetic valve degeneration and predict subsequent valve dysfunction, identifying patients at high risk of earlier valve failure.

**TRANSLATIONAL OUTLOOK:** Pragmatic studies are needed to define the role of ^18^F-fluoride PET-CT imaging in protocols for the surveillance of patients with bioprosthetic valves.
