# Supplementary material for: Detection and Prediction of Bioprosthetic Aortic Valve Degeneration
Source: J Am Coll Cardiol. 2019 Mar 19;73(10):1107–19. doi: 10.1016/j.jacc.2018.12.056 (PMC6424589; doi:10.1016/j.jacc.2018.12.056)
Supplement: Online Data [file mmc1.docx]

**SUPPLEMENTARY MATERIAL**

**Detection and Prediction of**

**Bioprosthetic Aortic Valve Degeneration**

Timothy RG Cartlidge, MD^1^, Mhairi K Doris, MD^1^, Stephanie L Sellers, PhD^3^, Tania A Pawade, MD^1^, Audrey C White^1^, Renzo Pessotto, MD^1^, Jacek Kwiecinski MD^1^, Alison Fletcher, PhD^2^, Carlos Alcaide, MSc^1^, Christophe Lucatelli, PhD^2^, Cameron Densem MD^4^, James HF Rudd, MD^5^, Edwin JR van Beek, MD^2^, Adriana Tavares, PhD^1^, Renu Virmani, MD^6^, Daniel Berman, MD^7^, Jonathon A Leipsic, MD^3^, David E Newby, DSc^1^, Marc R Dweck, PhD^1^

**Affiliations**:

^1^ British Heart Foundation Centre for Cardiovascular Science, University of Edinburgh, 49 Little France Crescent, Edinburgh, UK, EH16 4SB

^2^ Edinburgh Imaging Facility, Queen’s Medical Research Institute Facility, University of Edinburgh, 47 Little France Crescent, Edinburgh, UK, EH16 4SA

^3^Department of Radiology, St. Paul’s Hospital, University of British Columbia, Canada, V6Z 1Y6

^4^Department of Cardiology, Papworth Hospital NHS Foundation Trust, Cambridge, UK, CB23 3RE.

^5^Division of Cardiovascular Medicine, University of Cambridge, Cambridge, UK, CB2 2QQ

^6^CVPath Institute, Gaithersburg, Maryland, USA, 20878

^7^Cedars-Sinai Heart Institute, Los Angeles, California, USA, 90048

**SUPPLEMENTAL METHODS**

**Study Eligibility Criteria for Patient Selection**

*Inclusion Criteria*

Ability to give informed consent; over 40 years of age; Cohort 1: patients with a surgically implanted bioprosthetic aortic valve who are due to undergo redo-aortic valve surgery or valve-in-valve transcatheter valve implantation for bioprosthetic valve failure; Cohort 2: patients with a surgically implanted bioprosthetic aortic valve, implanted 1 month, 2 years, 5 years or 10 years prior to study recruitment and without known evidence of valve dysfunction or degeneration.

*Exclusion Criteria*

Inability to give informed consent; pregnancy; breastfeeding; claustrophobia; allergy to iodinated contrast; liver failure; chronic kidney disease (with estimated glomerular filtration rate <30 mL/min/1.73m^2^); Paget's disease; metastatic malignancy; inability to tolerate the supine position.

**Study Assessments and Data Collection**

*Echocardiography*

Two-dimensional and Doppler echocardiography was performed at baseline and annually for 2 years by a single experienced and British Society of Echocardiography-accredited echocardiographer using a single echocardiography scanner (Affiniti 70, Philips Healthcare) according to a standardized protocol. Aortic valve Doppler measurements were routinely assessed from the apex, suprasternal notch and right sternal edge and used to measure the peak velocity through the valve, the mean gradient and the Doppler velocity index (DVI). Mean values were taken from 3 measurements when subjects were in sinus rhythm and from 5 measurements if atrial fibrillation was present. Bioprosthetic valve regurgitation was graded as mild, moderate or severe according to guideline recommendation on the basis of visual appraisal of color Doppler images, measurement of pressure half-time (ms) and assessment for aortic flow reversal in diastole.(1,2) In cohort 2, all 71 patients completed baseline and 1 year echocardiographic assessments, with 67 completing the 2 year visit. Measurements of relevant continuous variables were assessed as an annualized change during the follow-up period.

*Defining Bioprosthetic Degeneration and Dysfunction*

Prosthetic valve dysfunction was adjudicated based upon European Association of Cardiovascular Imaging/Inter-American Society of Echocardiography/Brazilian Department of Cardiovascular Imaging and American Society of Echocardiography guidelines(1,2) using the following criteria: Doppler velocity index (DVI) <0.29 and either peak velocity >3 m/s or mean gradient >20 mmHg (i.e. valve dysfunction suggested by one flow-independent parameter and one flow-dependent parameter), prosthetic regurgitation of at least moderate severity or an increase in mean gradient >10 mmHg.(1,2)

We also investigated an alternative definition of bioprosthetic valve degeneration and dysfunction proposed in a recent expert consensus statement:(3) *stage 1* characterized by morphological abnormality (detected on echocardiography or computed tomography) in the absence of hemodynamic changes; *stage 2* characterized by the presence of either moderate valve obstruction, moderate regurgitation or both; stage 3 characterized by the presence of either severe valve obstruction or regurgitation. Central to this definition of structural valve degeneration is a deterioration in function over time, for example, new valve obstruction should be accompanied by a change in mean gradient >10mmHg associated with a decrease in Doppler velocity index and effective orifice area. Valve failure was defined as the development of severe stenosis or regurgitation resulting in the development of patient symptoms and/or the need for redo valve intervention.(4)

*Positron Emission Tomography and Computed Tomography*

Subjects were given 25 mg of oral metoprolol if their resting heart rate was >65 beats/min in the absence of any contraindication. A target dose of 125 MBq ^18^F-fluoride was administered intravenously, with an effective radiation dose of 3 mSv. After 60 min, participants were imaged on a hybrid 128-detector array positron emission tomography and computed tomography (PET-CT) scanner (Biograph mCT, Siemens, Erlangen, Germany). Pharmacokinetic modelling has demonstrated that this timing provides excellent vascular tissue contrast resolution.(5) A low-dose attenuation correction CT scan was performed (120 kV, 50 mAs; 5/3 mm), followed by acquisition of PET data in list mode, using a single 30-min bed position centered on the valve in 3-dimensional mode. Finally, electrocardiogram-gated aortic valve CT calcium scoring and contrast-enhanced CT angiography were performed in diastole and in held expiration.

**Image Analysis**

*^18^F-Fluoride* *PET-CT*

Static PET-CT images were reconstructed with correction applied for attenuation, dead time, scatter and random coincidences, using an optimized iterative reconstruction algorithm (ultra-HD; TrueX + TOF, matrix 200, zoom 1; Gaussian filter). Analyses were performed using an OsiriX workstation (OsiriX version 8.0.3 64-bit; OsiriX Imaging Software, Geneva, Switzerland). ECG-gated contrast-enhanced CT images were reconstructed in diastole using 1-mm slices. For this purpose, ECG-gating of list mode PET data was used to reconstruct at 25% intervals of the cardiac cycle with diastolic data determined as that acquired between 50 and 75% of the R-R interval. PET and CT reconstructions were reoriented, fused and carefully co-registered in all 3 planes using the 2-D orthogonal tool (Online Figure 2). Key points of reference were the sternum, vertebrae, blood-pool in the left ventricle (based upon the high ^18^F-fluoride activity in the blood relative to the surrounding myocardium), the ascending aorta and the aortic arch. CT scans were determined to be abnormal if there was evidence of circumferential pannus extending into the valve cusps, spotty leaflet calcification (<3 mm),(6) large leaflet calcification (≥3 mm) or non-calcific thickening (≥2 mm). PET scans were adjudicated to be abnormal if increased ^18^F-fluoride uptake (target-to-background ratio >1.3) originating in the valve cusps was observed. ^18^F-fluoride uptake originating from the sewing ring or the aortic wall was not considered as evidence of bioprosthetic leaflet degeneration and was not quantified.

*Quantification of ^18^F-Fluoride Uptake in Bioprosthetic Valve Leaflets*

To quantify ^18^F-fluoride uptake, a circular (area 1-cm^2^) region of interest (ROI) was drawn around the area of maximal uptake originating in the valve cusps on the reoriented co-registered PET-CT images, employing a ‘most diseased segment’ (MDS) approach.(7) Where there was no visible uptake in the valve cusps, a 1-cm^2^ circular ROI was drawn in the center of the valve. Mean and maximum standardized uptake values (SUV) were extracted from these ROIs and corrected for blood-pool activity measured in the right atrium (2-cm^2^ ROIs, axial slices, at the level of the right coronary ostium) to calculate the target-to-background ratio (TBR).(6) Mean TBR values using the MDS approach (TBR) were used as the principal comparator in the outcome analysis in keeping with data from native valve ^18^F-fluoride PET studies, although results for all PET measurements are presented.(7) The TBR>1.3 threshold was pre-selected based upon previous vascular ^18^F-fluoride studies and was supported by the threshold defining the upper tertile of bioprosthetic valve ^18^F-fluoride activity observed in this study.(8)

*Micro Positron Emission Tomography and Computed Tomography*

Explanted bioprosthetic valves were incubated with 2 MBq ^18^F-fluoride for 20 min and PET data were acquired over a 30-min bed time using 1:5 coincidence mode. Micro CT was then acquired (semi-circular full trajectory, maximum field of view, 720 projections, 70 kVp, exposure 300 ms and 1:1 binning) for attenuation correction and anatomical registration. PET data were reconstructed using Mediso’s iterative Tera-Tomo 3D algorithm and the following settings: 4 iterations, 6 subsets, full detector model, normal regularization, spike filter on, voxel size 0.6 mm, 400-600 keV energy window. Analysis was performed using PMOD (PMOD Technologies, Switzerland) to quantify calcium volume on CT and ^18^F-fluoride activity.

**Histopathology**

Explanted bioprosthetic valve specimens were fixed in 10% w/v buffered formalin phosphate (Fisher Chemical SF100-20) pending histological analysis. Heavily calcified samples were treated with fixative decalcifier to facilitate sectioning (Fisher Chemical, Cal-Ex II, CS511-1D). Leaflets were detached from the base of the valve frame and marked with tissue dye fixed with acetic acid to maintain orientation. Each leaflet was processed then sectioned into 2-4 mm sections and embedded in paraffin sequentially to yield 7-8 tissue cross-sections per leaflet. Paraffin sections (4 µm) were used for histology and immunohistochemistry (IHC). Tissue were sectioned and stained for architecture (hematoxylin and eosin or Movat’s pentachrome), calcium phosphate (von Kossa, Alizarin Red, alizarin red), thrombus and fibrosis (Movat Pentachrome), and collagen (Picrosirius Red).

**SUPPLEMENTARY RESULTS**

Patients in the highest tertile of ^18^F-fluoride uptake demonstrated greater disease progression than patients in the lower 2 tertiles (annualized change in valve peak velocity; tertile 1: 0.14 [0.05 to 0.30] *versus* tertiles 2 and 3: 0.01 [-0.02 to 0.10] m/s/year, p=0.006; Online Figure 3). The 4 patients with new or worsening prosthetic valve regurgitation during follow-up were also all amongst those in tertile 1.

Using the contemporary consensus definition,(3) bioprosthetic morphological abnormalities were observed in 9 patients in the absence of hemodynamic changes (stage 1). A further 5 patients had evidence of a significant deterioration in valve function: either an increase in mean gradient >10 mmHg from baseline post-operative echocardiography and moderate valve stenosis or an increase of at least 1 severity class of valvular regurgitation and at least moderate valvular regurgitation, or both (stage 2). Finally, two patients displayed rapid deterioration in valve function during follow-up with an increase in mean gradient >20 mmHg and severe valve stenosis (stage 3). ^18^F-Fluoride uptake was higher in patients with structural valve degeneration compared to patients without (TBR 1.54 [1.38-1.96] versus 1.08 [1.02-1.19], p<0.001).

**ONLINE FIGURE 1**

**CONSORT flow diagram of study recruitment, allocation, follow-up and analysis.**

**
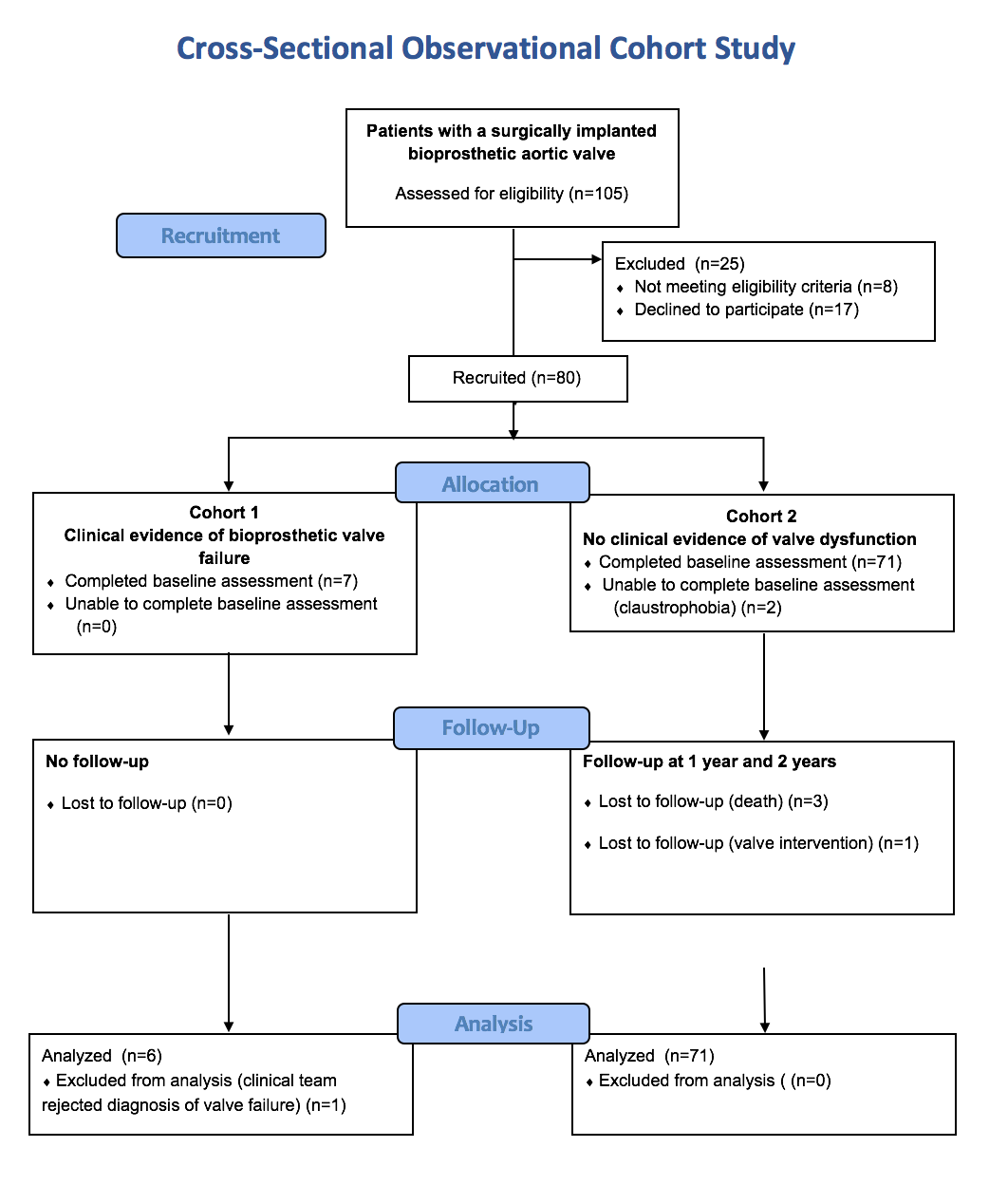
**

Flow diagram illustrating prospective study recruitment and allocation to pre-defined cross-sectional groups (Cohort 1: patients with suspected bioprosthetic valve failure, Cohort 2: patients under clinical review without evidence of valve dysfunction or degeneration grouped by time from valve implantation), follow-up and analysis with 77 volunteers included in the final analysis.

**ONLINE FIGURE 2**

**Methodology for co-registration of positron emission tomography and contrast computed tomography in bioprosthetic aortic valves.**

**
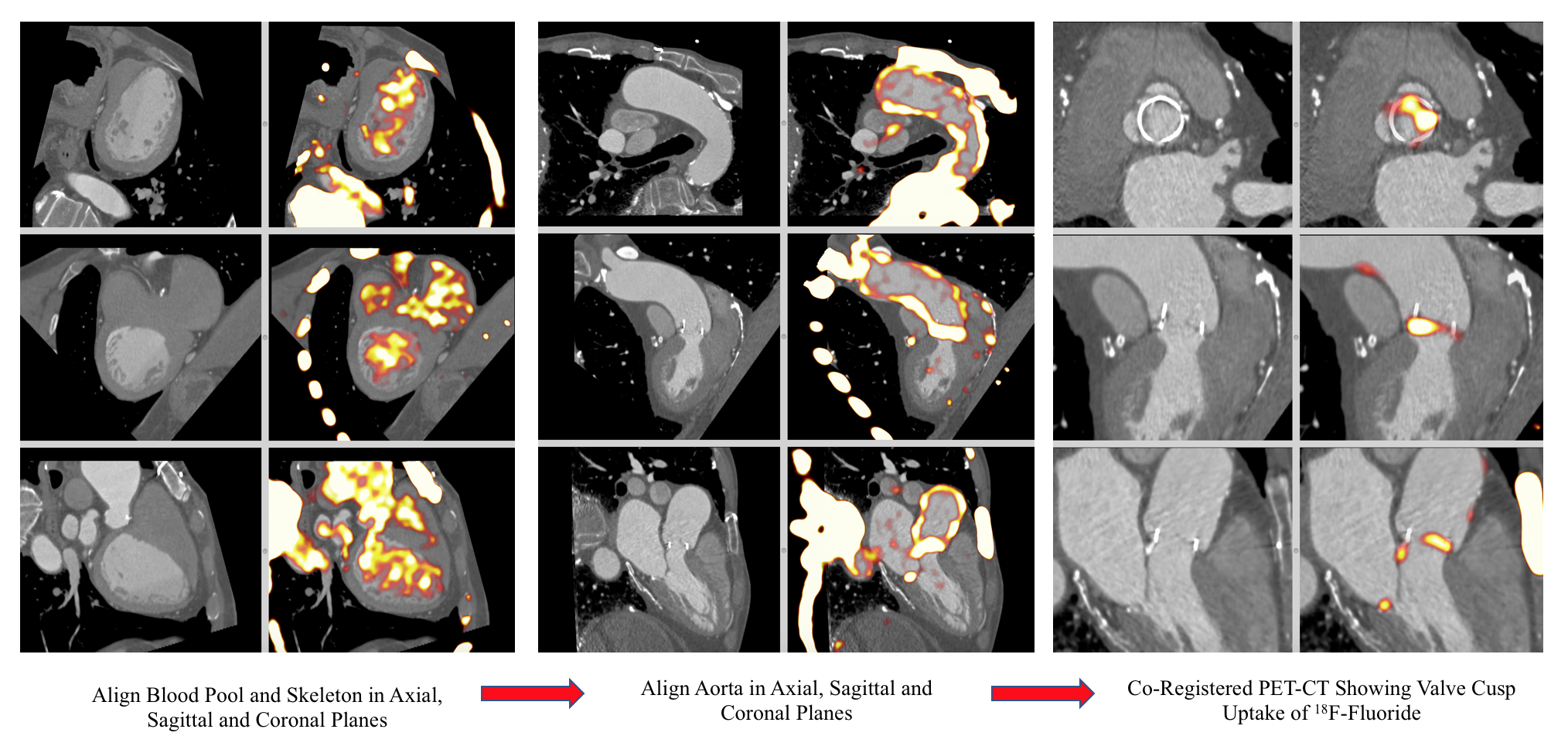
**

Careful alignment of the positron emission tomography (PET) activity in the cardiac blood pool with the contrast-enhanced computed tomography (CT) images is performed in 3 planes (left). This is possible because of the high 18F-fluoride activity in the blood pool compared to the myocardium. Subsequently PET activity in the ascending aorta and aortic arch (center) is aligned with the CT and final refinement of co-registration is performed according to landmarks around the bioprosthetic aortic valve such as the coronary arteries and mitral valve annulus. Hybrid images are then re-orientated to provide en face images of the bioprosthetic valve with corresponding perpendicular long-axis images. Finally, the PET windowing is adjusted to the point below which blood pool activity in the aorta is visible to assess uptake in the valve leaflets.

**ONLINE FIGURE 3**

**Tertile analysis according to ^18^F-fluoride PET uptake demonstrating tissue-to-background (TBR) values across the tertiles and relationship with change in valve function during follow-up.**

**
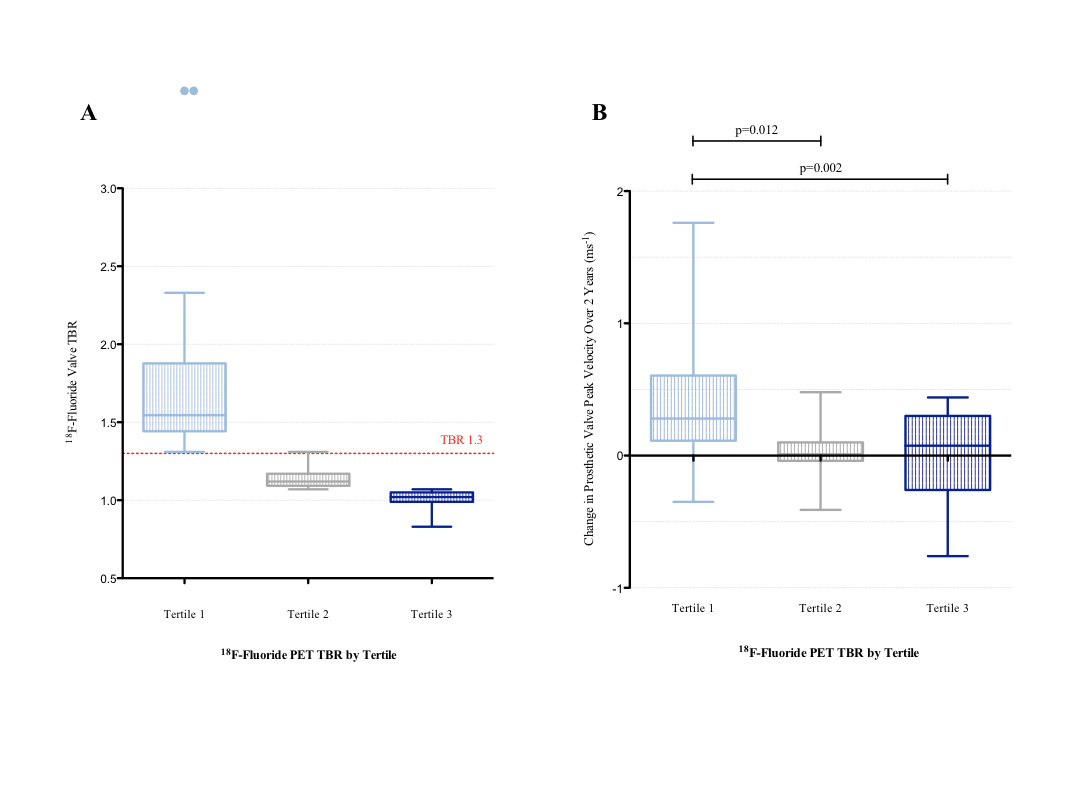
**

**A.** Box-plot illustrating the spread of ^18^F-fluoride tissue-to-background (TBR) values when grouped into tertiles. Dashed red line represents TBR 1.3 which was the cut off for the top tertile and also the threshold for discriminating increased uptake. **B**. Box-plot demonstrating haemodynamic progression (measured by the change in peak valve velocity between baseline and 2-year follow-up) when patients were grouped by tertile according to ^18^F-fluoride TBR values. Patients in tertile 1 (TBR >1.3) exhibited more rapid haemodynamic progression than those in the lower tertiles. The 4 patients with new or worsening prosthetic valve regurgitation during follow-up were also all amongst those in tertile 1.

**ONLINE TABLE 1**

**Summary statistics of effective radiation dose for baseline PET-CT using up-to-date PET effective dose conversion factor** 0.028 mSv·mGy^−1^cm^−1^ **(9)**

| **TOTAL RADIATION DOSES** | | | | | | |
| --- | --- | --- | --- | --- | --- | --- |
|  | **Number of subjects** | **Mean PET effective dose (mSv)** | **Mean CT effective dose (mSv)** | **Mean total effective dose (mSv)** | **Median total effective dose (mSv)** | **Expected effective dose (mSv)** |
| **Cohort 1** | 7 | 2.1 | 20.9 | 23.0 | 16.6 | 19.15 |
| **Cohort 2** | 71 | 2.2 | 13.4 | 15.6 | 13.9 | 19.15 |
| **All Subjects** | 78 | 2.1 | 14.2 | 16.4 | 14.2 | 19.15 |

**Abbreviations:** CT: computed tomography, PET: positron emission tomography.

**ONLINE TABLE 2**

**The different types of bioprosthetic valve models in the clinical *in-vivo* cohorts.**

Number of valves in each group shown.

Cohort 1: patients with degenerated and failing bioprosthetic valves.

Cohort 2: patients without previously established bioprosthetic valve degeneration grouped according to duration of implantation.

| **MODEL OF BIOPROSTHETIC AORTIC VALVE** | | | | | | |
| --- | --- | --- | --- | --- | --- | --- |
|  | **COHORT 1** | **COHORT 2** | | | | |
|  |  | **TOTAL** | **1 MONTH** | **2 YEAR** | **5 YEAR** | **10 YEAR** |
| **St Jude Biocor**  **(Porcine Stented)** | 0 | 1 | 0 | 0 | 0 | 1 |
| **Carpentier-Edwards (Porcine Stented)** | 1 | 8 | 0 | 0 | 3 | 5 |
| **Carpentier-Edwards Perimount**  **(Pericardial Stented)** | 1 | 50 | 9 | 22 | 13 | 6 |
| **Medtronic Mosaic**  **(Porcine Stented)** | 0 | 2 | 0 | 0 | 0 | 2 |
| **Aortech**  **(Polymeric Stented)** | 0 | 1 | 0 | 0 | 0 | 1 |
| **Medtronic Hancock (Porcine Stented)** | 0 | 1 | 0 | 0 | 0 | 1 |
| **Vascutek Aspire**  **(Porcine Stented)** | 0 | 3 | 0 | 0 | 0 | 3 |
| **Sorin Mitroflow (Pericardial Stented)** | 1 | 4 | 0 | 0 | 4 | 0 |
| **Edwards Prima**  **(Porcine Stentless)** | 0 | 1 | 0 | 0 | 0 | 1 |
| **Vascutek Elan**  **(Porcine Stentless)** | 1 | 0 | 0 | 0 | 0 | 0 |
| **St. Jude Toronto**  **(Porcine Stentless)** | 1 | 0 | 0 | 0 | 0 | 0 |
| **Medtronic Freestyle (Porcine Stentless)** | 1 | 0 | 0 | 0 | 0 | 0 |

**ONLINE TABLE 3**

**Bioprosthetic valve model size in the clinical *in-vivo* cohorts**

Cohort 1: patients with degenerated and failing bioprosthetic valves.

Cohort 2: patients without previously established bioprosthetic valve degeneration grouped according to duration of implantation. Number of valves in each group shown.

| **SIZE OF BIOPROSTHETIC AORTIC VALVE** | | | | | | | |
| --- | --- | --- | --- | --- | --- | --- | --- |
|  | **19mm** | **21mm** | **23mm** | **25mm** | **27mm** | **29mm** |  |
| **St Jude Biocor**  **(Porcine Stented)** | 0 | 0 | 1 | 0 | 0 | 0 |  |
| **Carpentier-Edwards (Porcine Stented)** | 1 | 0 | 5 | 2 | 0 | 1 |  |
| **Carpentier-Edwards Perimount**  **(Stented Pericardial)** | 6 | 11 | 19 | 13 | 3 | 0 |  |
| **Medtronic Mosaic**  **(Porcine Stented)** | 1 | 0 | 1 | 0 | 0 | 0 |  |
| **Aortech**  **(Polymeric Stented)** | 0 | 0 | 0 | 0 | 1 | 0 |  |
| **Medtronic Hancock (Porcine Stented)** | 0 | 0 | 0 | 1 | 0 | 0 |  |
| **Vascutek Aspire**  **(Porcine Stented)** | 0 | 1 | 1 | 0 | 1 | 0 |  |
| **Sorin Mitroflow (Pericardial Stented)** | 1 | 1 | 2 | 1 | 0 | 0 |  |
| **Edwards Prima Plus**  **(Porcine Stentless)** | 0 | 1 | 0 | 0 | 0 | 0 |  |
| **Vascutek Elan**  **(Porcine Stentless)** | 0 | 0 | 0 | 1 | 0 | 0 |  |
| **Medtronic Freestyle (Porcine Stentless)** | 0 | 0 | 0 | 1 | 0 | 0 |  |
| **Total** | **9** | **14** | **29** | **19** | **5** | **1** |  |

**ONLINE TABLE 4**

**Factors associated with deterioration in bioprosthetic valve function (annualized change in peak velocity after 2 years): univariable analysis**

| **UNIVARIABLE ANALYSIS OF PREDICTORS OF PROGRESSION IN PEAK VELOCITY** | | | | |
| --- | --- | --- | --- | --- |
|  | **Unstandardized Coefficient**  **(95% CI)** | **Standard Error** | **Standardized Coefficient** | **Significance** |
| **Sex** | 0·056  (-0·042 to 0·154) | 0·049 | 0·138 | 0·256 |
| **Age** | 0·001  (-0·006 to 0·008) | 0·004 | 0·039 | 0·752 |
| **BMI** | -0·003  (-0·014 to 0·008) | 0·005 | -0·069 | 0·569 |
| **BSA** | -0·096  (-0·308 to 0·115) | 0·106 | -0·110 | 0·366 |
| **Valve Age** | 0·003  (-0·010 to 0·016) | 0·007 | 0·050 | 0·683 |
| **Valve Type** | -0·011  (-0·045 to 0·024) | 0·017 | -0·075 | 0·539 |
| **Systolic BP** | 0·001  (-0·001 to 0·003) | 0·001 | 0·125 | 0·301 |
| **Hypertension** | -0·073  (-0·178 to 0·032) | 0·052 | -0·167 | 0·168 |
| **Diabetes** | -0·016  (-0·207 to 0·175) | 0·096 | -0·020 | 0·867 |
| **Dyslipidemia** | -0·004  (-0·121 to 0·113) | 0·059 | -0·009 | 0·944 |
| **Smoking** | 0·153  (0·002 to 0·303) | 0·075 | 0·239 | **0·047*** |
| **Anticoagulation** | -0·119  (-0·329 to 0·091) | 0·105 | -0·136 | 0·263 |
| **ACEi or ARB** | -0·024  (-0·123 to 0·074) | 0·049 | -0·060 | 0·625 |
| **Statin** | -0·028  (-0·135 to 0·079) | 0·054 | -0·063 | 0·604 |
| **Baseline Peak Velocity** | 0·001  (-0·095 to 0·098) | 0·048 | 0·003 | 0·978 |
| **Abnormal CT** | 0·102  (-0·020 to 0·223) | 0·061 | 0·200 | 0·099 |
| **Calcification on CT** | 0·047  (-0·129 to 0·222) | 0·088 | 0·064 | 0·597 |
| **NCLT on CT** | 0·142  (-0·046 to 0·330) | 0·094 | 0·179 | 0·137 |
| **Pannus on CT** | 0·068  (-0·095 to 0·231) | 0·082 | 0·100 | 0·409 |
| **^18^F-Fluoride Uptake (categorical)** | 0·184  (0·090 to 0·278) | 0·047 | 0·429 | **<0·001*** |
| **PET SUV _MDS mean_** | 0·383  (0·253 to 0·514) | 0·065 | 0·579 | **<0·001*** |
| **PET SUV _MDS max_** | 0·404  (0·280 to 0·527) | 0·062 | 0·620 | **<0·001*** |
| **PET TBR _MDS mean_** | 0·499  (0·383 to 0·615) | 0·058 | 0·720 | **<0·001*** |
| **PET TBR _MDS max_** | 0·466  (0·357 to 0·575) | 0·055 | 0·718 | **<0·001*** |

**Abbreviations:** ACEi: angiotensin-converting enzyme inhibitor, ARB: angiotensin receptor blockade, MDS: most diseased segment, PET: positron emission tomography, SUV: standardized uptake value, TBR: target to background ratio.

**ONLINE TABLE 5**

**A. Factors associated with deterioration in bioprosthetic valve function (annualized change in peak velocity after 2 years): multivariable analysis using ^18^F-fluoride PET as a continuous variable quantified by TBR.**

| **MULTIVARIABLE LINEAR REGRESSION MODEL 1:**  **PREDICTORS OF ANNUALIZED CHANGE IN PEAK TRANSVALVULAR VELOCITY**  **(PET AS CONTINUOUS VARIABLE)** | | | | |
| --- | --- | --- | --- | --- |
| **SUMMARY: R = 0·753 R Square 0·567 Std· Error 0·142 p <0·001** | | | | |
| **Variable** | **Unstandardized**  **Coefficient (95% CI)** | **Standard Error** | **Standardized Coefficient** | **Significance** |
| **Age** | 0·000 (-0·005-0·006) | 0·003 | 0·011 | 0·904 |
| **Sex** | 0·032 (-0·043-0·107) | 0·038 | 0·079 | 0·396 |
| **Valve Age** | -0·010 (-0·020-0·000) | 0·005 | -0·187 | 0·051 |
| **Baseline Peak Velocity** | -0·022 (-0·097-0·053) | 0·037 | -0·055 | 0·556 |
| **Abnormal CT** | 0·030 (-0·131-0·071) | 0·050 | -0·059 | 0·552 |
| **PET TBR** | 0·562 (0·432-0·693) | 0·065 | 0·801 | **<0·001*** |

**Abbreviations:** CT: computed tomography, PET: positron emission tomography, TBR: target to background ratio.

**B. Factors associated with deterioration in bioprosthetic valve function (annualized change in peak velocity after 2 years): multivariable analysis using ^18^F-fluoride PET as a dichotomous variable (either normal or abnormal).**

| **MULTIVARIABLE LINEAR REGRESSION MODEL 2:**  **PREDICTORS OF ANNUALIZED CHANGE IN PEAK TRANSVALVULAR VELOCITY**  **(PET AS CATEGORICAL VARIABLE)** | | | | |
| --- | --- | --- | --- | --- |
| **SUMMARY: R = 0·453 R Square 0·205 Std· Error 0·192 p = 0·023** | | | | |
| **Variable** | **Unstandardized Coefficient (95% CI)** | **Standard Error** | **Standardized Coefficient** | **Significance** |
| **Age** | 0·001 (-0·006-0·009) | 0·004 | 0·047 | 0·703 |
| **Sex** | 0·062 (-0·041-0·164) | 0·051 | 0·15 | 0·234 |
| **Valve Age** | -0·003 (-0·016-0·011) | 0·007 | -0·050 | 0·690 |
| **Baseline Peak Velocity** | -0·010 (-0·112-0·092) | 0·051 | -0·025 | 0·844 |
| **Abnormal CT** | -0·043 (-0·197-0·111) | 0·077 | -0·084 | 0·581 |
| **Abnormal PET** | 0·205 (0·086-0·324) | 0·059 | 0·477 | **0·001*** |

**Abbreviations:** CT: computed tomography, PET: positron emission tomography, TBR: target to background ratio.

**ONLINE TABLE 6**

**Factors associated with the development of bioprosthetic valve dysfunction as a dichotomous variable according to expert consensus statement(3): binary logistic regression using ^18^F-fluoride PET as a continuous variable quantified by TBR.**

| **BINARY LOGISTIC REGRESSION:**  **PREDICTORS OF NEW STRUCURAL VALVE DEGENERATION**  **(PET AS CONTINUOUS VARIABLE)** | | | | |
| --- | --- | --- | --- | --- |
| **Variable** | **Unstandardized Coefficient** | **Standard Error** | **Odds Ratio**  **(95% CI)** | **Significance** |
| **Age** | 0.189 | 0.128 | 1.21  (0.94-1.56) | 0.140 |
| **Sex** | 0.956 | 1.574 | 2.60  (0.12-56.90) | 0.544 |
| **Valve Age** | -0.263 | 0.283 | 0.77  (0.44-1.34) | 0.353 |
| **Baseline Peak Velocity** | 5.138 | 2.435 | 170.33  (1.44-20131.76) | **0.035*** |
| **Abnormal CT** | 0.663 | 1.551 | 1.94  (0.09-40.58) | 0.669 |
| **PET TBR** | 6.814 | 2.924 | 910.52  (2.952-280843.31) | **0.020*** |

**Abbreviations:** CT: computed tomography, PET: positron emission tomography, TBR: target to background ratio.

**Factors associated with the development of bioprosthetic valve dysfunction as a dichotomous variable according to echocardiography guidelines(1,2): binary logistic regression using ^18^F-fluoride PET as a continuous variable quantified by TBR.**

| **BINARY LOGISTIC REGRESSION:**  **PREDICTORS OF NEW STRUCURAL VALVE DEGENERATION**  **(PET AS CONTINUOUS VARIABLE)** | | | | |
| --- | --- | --- | --- | --- |
| **Variable** | **Unstandardized Coefficient** | **Standard Error** | **Odds Ratio**  **(95% CI)** | **Significance** |
| **Age** | -0.043 | 0.108 | 0.958  (0.775-1.183) | 0.689 |
| **Sex** | -0.936 | 1.321 | 0.392  (0.029-5.221) | 0.479 |
| **Valve Age** | -0.105 | 0.229 | 0.901  (0.575-1.411) | 0.648 |
| **Baseline Peak Velocity** | 3.387 | 1.652 | 29.567  (1.159-754.013) | **0.040*** |
| **Abnormal CT** | -0.669 | 1.511 | 0.512  (0.026-9.908) | 0.658 |
| **PET TBR** | 7.572 | 2.662 | 1942.81  (10.53-358307.91) | **0.004*** |

**REFERENCES**

1. Lancellotti P, Pibarot P, Chambers J, et al. Recommendations for the imaging assessment of prosthetic heart valves: a report from the European Association of Cardiovascular Imaging endorsed by the Chinese Society of Echocardiography, the Inter-American Society of Echocardiography, and the Brazilian Department of Cardiovascular Imaging. *Eur Heart J-Card Img* 2016;**17**(6):589-590.
2. Zoghbi WA, Chambers JB, Dumesnil JG, et al. Recommendations for Evaluation of Prosthetic Valves With Echocardiography and Doppler Ultrasound. *J Am Soc Echocardiog* 2009;**22**(9):975–1014.
3. Dvir D, Bourguignon T, Otto CM, et al. Standardized Definition of Structural Valve Degeneration for Surgical and Transcatheter Bioprosthetic Valves. *Circulation* 2018;**137**(4):388-399.
4. Capodanno D, Petronio AS, Prendergast B et al. Standardized definitions of structural deterioration and valve failure in assessing long-term durability of transcatheter and surgical aortic bioprosthetic valves: a consensus statement from the European Association of Percutaneous Cardiovascular Interventions (EAPCI) endorsed by the European Society of Cardiology (ESC) and the European Association for Cardio-Thoracic Surgery (EACTS). Eur Heart J 2017;38:3382-3390.
5. Irkle A, Vesey AT, Lewis DY, et al. Identifying active vascular microcalcification by (18)F-sodium fluoride positron emission tomography. *Nat Comm*. 2015;**6**:7495.
6. Motoyama S, Kondo T, Sarai M, et al. Multislice computed tomographic characteristics of coronary lesions in acute coronary syndromes. *J Am Coll Cardiol* 2007;50:319-326.
7. Pawade TA, Cartlidge TR, Jenkins WS, et al. Optimization and Reproducibility of Aortic Valve 18F-Fluoride Positron Emission Tomography in Patients with Aortic Stenosis. *Circ-Cardiovasc Imag*. 2016;**9**(10):e005131.
8. Joshi NV, Vesey AT, Williams MC, et al. 18F-Fluoride positron emission tomography for identification of ruptured and high-risk coronary atherosclerotic plaques: a prospective clinical trial. *Lancet* 2014;**383**:705–713.
9. Administration of Radioactive Substances Advisory Committee. ARSAC notes for guidance: good clinical practice in nuclear medicine 2017;appendix 1 part B, accessed 8 Nov 2018, <<https://www.gov.uk/government/publications/arsac-notes-for-guidance>>
